# A population-based cross-sectional study of age-specific risk factors for high risk human papillomavirus prevalence in rural Nigeria

**DOI:** 10.1186/1750-9378-6-12

**Published:** 2011-07-29

**Authors:** Megan A Clarke, Julia C Gage, Kayode O Ajenifuja, Nicolas A Wentzensen, Akinfolarin C Adepiti, Sholom Wacholder, Robert D Burk, Mark Schiffman

**Affiliations:** 1 Division of Cancer Epidemiology and Genetics, National Cancer Institute, National Institutes of Health, DHHS, Bethesda, MD, USA; 2 Department of Obstetrics, Gynaecology & Perinatology, Obafemi Awolowo University, Ile-Ife, Nigeria; 3 Departments of Microbiology and Immunology, Pediatrics, Obstetrics, Gynecology, and Women's Health, and Epidemiology and Population Health, Albert Einstein Cancer Center, Albert Einstein College of Medicine, Bronx, New York, USA

## Abstract

**Background:**

Cervical cancer, caused by persistent infection with carcinogenic human papillomavirus (HR-HPV), is particularly prevalent in Sub-Saharan Africa and is associated with a high mortality rate. Some studies in West Africa, including our own, have found unusually high HR-HPV across all ages with a slight peak in older women. This increased prevalence at older ages may complicate screen-and-treat programs, which are implemented in regions where HPV prevalence declines with age and typically target women between 30-49 years. A better understanding of the determinants of high HR-HPV prevalence at older ages is needed. The goal of this study is to explore risk factors for HR-HPV prevalence by age among women in our population-based study in Irun, a rural town in southwestern Nigeria.

**Methods:**

1,420 women were administered a clinic-based questionnaire regarding sexual and reproductive behavior, marital status (including co-wives), and malaria exposure. Logistic regression compared questionnaire responses and PCR positivity for a set of 13 carcinogenic HR-HPV types. Results were stratified by age (15-29, 30-45, 46-55, and 56+ years).

**Results:**

Birth control use and age at first pregnancy were associated with HR-HPV (*p-value *= 0.03 and 0.05, respectively). Early age at sexual debut and multiple sex partners were risks for HR-HPV, but did not reach significance (*p-value *= 0.1 and 0.07, respectively). Neither self-reported malaria nor presence of co-wives in the household was associated with HR-HPV (*p-value *= 0.85 and 0.24, respectively). In age sub-categories, early age at sexual debut was a significant risk factor for HR-HPV among women 35-45 years (*p-value = 0.02*). Early age at first pregnancy remained a significant risk factor for women aged 56+ years (*p-value *= 0.04). Greater than 2 sex partners and use of birth control were associated (though not significantly) with HR-HPV in women aged 30-45 (*p-value *= 0.08, respectively).

**Conclusions:**

In this high-risk region with elevated HR-HPV prevalence at older ages, we confirmed previously described, behavioral determinants of HR-HPV. There was no association with self-reported malaria or co-wives, which we had hypothesized might correlate with HR-HPV at older ages.

## Background

Cervical cancer, caused by persistent infection with carcinogenic human papillomavirus (HR-HPV), is the second most common cancer in women worldwide and the leading cause of cancer deaths among women in developing countries [1]. Cervical cancer is particularly prevalent in Sub-Saharan Africa and is associated with a high mortality rate [1-3]. Reasons for this increased prevalence are not completely understood, but likely include limited access to medical care (especially in rural areas) and lack of available or affordable treatment options [4]. Other geographic and cultural risk factors that are widespread in Sub-Saharan Africa include: early marriage, polygamous marriages, and high parity. Immuno-compromising infections such as the Human Immunodeficiency Virus (HIV) and chronic malaria are also endemic in this region [5,6].

The age-specific pattern of HR-HPV prevalence can differ somewhat by geographic location. In most populations, incidence and prevalence of HR-HPV infection peak at young ages soon after sexual debut, followed by a decline as nearly all infections clear and new sexual encounters decrease [7,8]. This pattern motivates the use of HR-HPV screen-and-treat programs, targeted to women past the peak age of HR-HPV prevalence.

However, high HR-HPV prevalence at all ages has been reported in some, but not all, population-based studies conducted in sub-Saharan Africa, including areas of West Africa [9-11]. Recently, we have shown a similar pattern from our population-based study in Irun, a rural village in southwestern state of Ondo, Nigeria, where the prevalence of HR-HPV even among cervical cytologically normal women appears to be consistently elevated across all ages with a slight peak in older women [12]. The reasons for the high HR-HPV prevalence at older ages, which may negatively affect the performance of HR-HPV screen-and-treat programs, are unknown. Data on HR-HPV risk factors in among older women, particularly in West Africa, are very limited [13-15].

Here we present a study examining clinic-based questionnaire data from approximately 1,400 women in our population-based cohort in Irun, Nigeria. The goal of this study was to evaluate the association between known and possible, novel risk factors for HR-HPV infection such as sexual and reproductive behaviors, living in a household with co-wives, and self-reported malaria exposure. Since we are particularly interested in understanding the elevated HR-HPV prevalence among older women in this region, we extended our analyses to look at these risk factors among women of different age groups.

## Methods

The protocol was reviewed and approved by both Nigerian and NCI institutional review boards. Detailed methodologic aspects of this study are described elsewhere [12]. Briefly, households were surveyed based on a census done by local health workers. All houses known to have a household with co-wives as well as a random sample of the remaining houses (439 total) were selected to reach approximately 2,100 women. Women were screened in their homes by a local health worker for eligibility criteria (not pregnant, without a hysterectomy, 15+ years of age, lived in the house for more than three months). A total of 2,091 women were deemed eligible for enrollment. About one-third (n = 669) refused enrollment either at home or did not show up for clinic. Participation varied by age: women aged 15-20 years were less likely to enroll and attend clinic visit (43.1% vs. 74.3% among women over 20). A total of 1,422 women attended a clinic appointment.

At the clinic visit, women completed a second informed consent and were given more detailed information of clinic procedures. Following consent, 1,420 women were administered a questionnaire that addressed potential risk factors associated with HR-HPV and cervical pre-cancer such as tar and tobacco exposure, number of malaria diagnoses within the past two years, menstrual history, sexual behavior, marital status, and birth control use. Nurses conducted a cervical exam involving the collection of cervical cells using a broom device and endocervical brush, and placed into PreservCyt buffer. On the day of collection, 1 ml was removed, frozen, and subsequently used for the HR-HPV DNA testing discussed here.

The presence of HR-HPV DNA was determined from the 1 ml of residual cytology specimens using MY09-MY11 PCR-based method as described [16]. We considered 13 HPV types to be carcinogenic types: 16, 18, 31, 33, 35, 39, 45, 51, 52, 56, 58, 59, and 68. Of 1,282 non-virgins for whom HPV DNA test results were available, 14.7% were infected with one or more carcinogenic HR-HPV genotypes [12].

Based upon previous age analysis of HR-HPV in this population of women and taking menopausal status into account [12], age categories were defined as follows: 15-29 years (n = 300), 30-45 years (n = 452), 46-55 years (n = 238) and 56+ years (n = 430). Logistic regression was used to estimate odds ratios (ORs) and corresponding 95% confidence intervals (95% CI) for risk factors of testing PCR positive for at least one of 13 carcinogenic HR-HPV types among all women and stratified by age. Questionnaire variables were categorized based on observed trends in the data. P-values are reported as p-trend when variables with more than two categories were analyzed. To explore residual confounding by age, within each age strata we adjusted for age in the logistic regressions and did not find any significant differences. To rule out the possibility that our findings were biased by oversampling women who lived in houses containing a household with more than one co-wife, we stratified our results by co-wife status in the household (no co-wife vs. one or more co-wives) and found no notable differences. Analyses were performed using Stata 11.0 analytic software (Stata Corp LP, College Station, TX).

## Results

**Figure 1:**
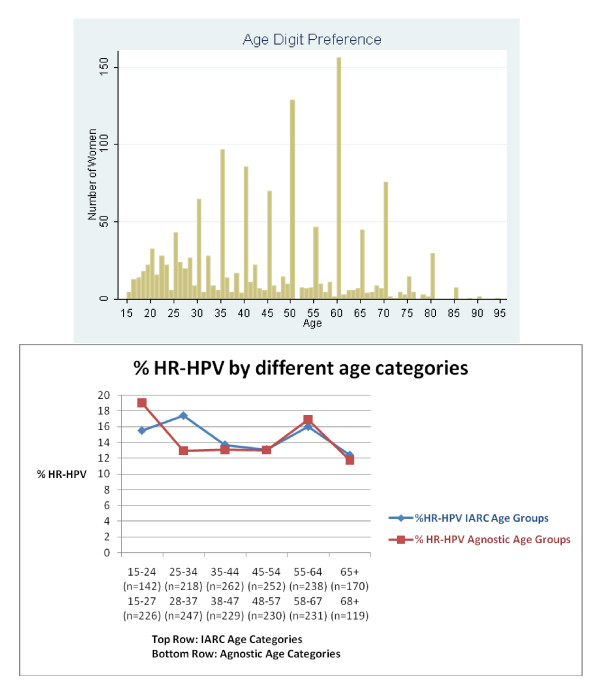
**Top: Graph showing preference for terminal digits 0 and 5 for self-reported age**. Bottom: Percentage of women with high-risk human papillomavirus (HR-HPV) shown using two different strategies for categorizing age. Abbreviations: IARC, International Agency for Research on Cancer.

The interviewers reported that the overall quality of the interview was either generally reliable (75.8%) or of high quality (22.2%). However, as shown in Figure 1, we observed a tendency for women to respond with preferences for terminal digits, e.g., of '0' or '5' when reporting their age. To verify the accuracy of our most important age analyses, we analyzed HR-HPV prevalence by using the standard IARC age categories [17] and by a more agnostic age stratification in which women were grouped according to the midpoint between digit preferences. Using these two different age categories, we found slightly different HR-HPV prevalence curves among younger women (Figure 1). We attribute this difference to the high HR-HPV prevalence among women aged 25 years (36%). In contrast, the curve appeared similar among older women.

Table 1 summarizes the clinical questionnaire data and shows the relationship between these risk factors and HR-HPV positivity. In terms of menstrual history, most women (70%) reported age of menarche to be between 15-19 years (average age 16.1 ± 2.5 years). The majority of women reported an average age at sexual debut of 20 years. Most women reported only one sex partner in the last two years (~80%). The average age of first pregnancy was 23.1 years and about 50% of women reported having between 5-9 lifetime pregnancies (average number 6.5 ± 3.2 pregnancies). Only a small percentage of women reported using birth control (14%) and of those women very few responded as to what type, although hormonal contraception appeared to be the predominant method in comparison to condom use (data not shown).

**Table 1:** Clinical questionnaire responses and potential risk factors for high risk human papillomavirus positivity

		Total		Positive for one or more HR-HPV genotypes				
	**Categories**	**n**	**(%)**	**n**	**(%)**	**OR**	**95% CI**	**p-value^c^**

**Age (yrs)** (mean = 45.2 ± 17, median = 45)	15 - 29	300	21.1	49	18.9	1.3	0.9 - 2.0	
Mean = 45.2, median = 45	30 - 45	452	31.8	55	12.8	0.8	0.6 - 1.2	
	46 - 55	238	16.8	29	12.7	0.8	0.5 - 1.3	
	56+	430	30.3	55	15.1	1.0	Ref	0.14

**Age of Menarche (yrs)**	10 - 14	224	17.6	33	16.1	1.0	0.6 - 1.8	
Mean = 16.1, median = 15	15 - 19	891	70.0	119	14.6	0.9	0.6 - 1.5	
	20 - 25	158	12.4	22	15.9	1.0	Ref	0.83

**Age at Sexual Debut (yrs)**	10-14	47	3.5	12	25.5	2.4	1.1 - 5.3	
Mean = 20.0, median = 20	15-24	1,059	79.7	148	14.8	1.2	0.8 - 2.0	
	25+	223	16.8	23	12.2	1.0	Ref	0.10

**Age at First Pregnancy (yrs)**	12-19	178	14.4	27	16.2	1.4	0.9 - 2.4	
Mean = 23.1, median = 23	20-24	523	42.4	84	17.1	1.5	1.1 - 2.2	
	25+	534	43.2	56	11.8	1.0	Ref	0.05

**Lifetime No. of Pregnancies**	1 - 4	370	28.9	54	15.7	1.1	0.7 - 1.8	
Mean = 6.5, median = 6	5 - 9	660	51.5	88	14.5	1.0	0.7 - 1.6	
	10+	251	19.6	32	14.2	1.0	Ref	0.86

**No. of Sex Partners (past 2 yrs)**	2+	311	23.1	54	18.2	1.4	1.0 - 2.0	
Mean = 1.3, median = 1	1	1,034	76.9	131	13.9	1.0	Ref	0.07

**Current Birth Control Use**	Yes	185	13.9	37	20.3	1.6	1.1 - 2.4	
	No	1,147	86.1	144	13.9	1.0	Ref	0.03

**Current Marital Status^a^**	Not in Union	450	33.5	66	16.4	1.2	0.9 - 1.7	
	In Union	895	66.5	117	13.9	1.0	Ref	0.25

**In Household with Co-wives**	Yes	553	40.1	70	13.5	0.8	0.6 - 1.1	0.24
	No	825	59.9	116	15.9	1.0	Ref	

**No. Other Wives**	1	185	37.2	29	16.4	1.3	0.7 - 2.4	
Mean = 2.1, median = 2	2	168	33.8	14	9.0	0.6	0.3 - 1.4	
	3+	144	29.0	18	13.3	1.0	Ref	0.13

**Rank Among Other Wives**	1^st^	222	45.0	26	12.4	1.3	0.6 - 2.9	
Mean = 1.8, median = 2	2^nd^	172	34.9	25	15.5	1.7	0.8 - 3.8	
	3^rd^	99	20.1	9	9.8	1.0	Ref	0.40

**Age of Youngest Wife (yrs)**	15 - 29	42	13.9	8	19.1	0.7	0.2 - 2.2	
Mean = 39.4, median = 40	30 - 59	236	78.1	25	11.4	0.4	0.1 - 1.0	
	60+	24	8.0	6	26.1	1.0	Ref	0.11

**Tobacco Exposure^b^**	Yes	121	15.2	20	17.5	1.3	0.8 - 2.3	
	No	675	84.8	87	13.8	1.0	Ref	0.31

**Number of Malaria Diagnoses (past 2 yrs)**	3+	453	35.2	64	15.5	1.1	0.6 - 2.1	
	2	371	28.9	45	13.4	0.9	0.5 - 1.8	
Mean = 2.2, median = 2	1	355	27.6	43	13.7	0.9	0.5 - 1.8	
	0	107	8.3	14	14.3	1.0	Ref	0.85

a - In Union includes a woman who is either married or living with a man;

b -A "yes" response includes 77 women whose husbands smoke and 44 women who currently use tobacco

c - p-trend for variables with more than two response categories.

In terms of marital status and related variables, the majority of women were in union (either married or living with a man) and about 60% of these women reported that they resided in a household with co-wives, reflecting the over-sampling in our field effort. A relatively equal percentage reported having 1, 2, or 3+ co-wives and 45% stated that they were ranked first among other wives. This reflects that our recruitment efforts were particularly successful among older women. The average age of the youngest wife was 39.4 ± 10.7 years.

Most women reported that neither they nor their husbands used tobacco, thus the overall exposure to tobacco was relatively low (15.2%). A majority of women reported having at least one case of malaria in the past two years (average number of diagnoses 2.2 ± 1.5).

Overall, age was observed to be a risk factor, although a non-linear one, for presence of HR-HPV, as we observed an increased prevalence in women 15-29 (18.9%) and 56+ years (15.1%). An age of 10-14 years at sexual debut was associated with a nearly two and a half-fold risk for HR-HPV infection (OR 2.4; 95% CI 1.1-5.3) compared to late age. Early rather than late age of first pregnancy was also associated with increased HR-HPV prevalence with a 40% increased risk for ages 12-19 (OR 1.4; 95% CI 0.9-2.4) and a 50% increased risk for ages 20-24 years (OR 1.5; 95% CI 1.1-2.2) compared to late age. Having more than two sexual partners and use of birth control were also positively related to HR-HPV risk (OR 1.4; *p-value *= 0.07 and OR 1.6; *p-value *= 0.03).

With regard to our more novel hypothesized risk factors, we did not find associations of interest. Living in a household with co-wives was associated with a slightly decreased risk of HR-HPV positivity (OR 0.8; *p-value *= 0.24) although this relationship was not significant. The number of self-reported malaria diagnoses in the past two years was not associated with increased risk of HR-HPV prevalence (*p-value *= 0.85).

Table 2 shows the distribution of key questionnaire variables determined by the analyses above, including those that we felt to be important risk factors to explore, stratified by age. Women 15-29 years reported younger ages at sexual debut and first pregnancy while women 50+ years recalled the highest ages for these measures. In addition, younger women were more likely to have greater than two sex partners in comparison to women aged 45-55 and 56+ years. The number of women using birth control was highest among the 46-55 year olds and lowest in women aged 56+ years. Women 30 years or older were the most likely to be in a household with co-wives, while most women aged 15-29 years did not live in a household with co-wives. Interestingly, self-reported malaria was most prevalent in women aged 56+ years, who were more likely to report three or more cases in the past 2 years and lowest among women 15-29 years of age, who frequently reported one or fewer cases.

**Table 2:** Key clinical questionnaire responses stratified by age

	15-29 Years		30-45 Years		46-55 Years		56+ Years		p-value^a^
	**n**	**Row %**	**n**	**Row %**	**n**	**Row %**	**n**	**Row %**	

**Age of Sexual Debut (yrs)**									< 0.01
**10-14**	20	7.7	19	4.4	7	3.1	1	0.3	
**15-24**	235	90.7	379	87.9	174	77.7	274	66.0	
**25+**	4	1.6	33	7.7	43	19.2	140	33.7	

**Age First Pregnancy (yrs)**									< 0.01
**12-19**	73	38.0	57	13.4	28	12.7	20	5.1	
**20-24**	105	54.7	219	51.3	80	36.4	119	30.0	
**25+**	14	7.3	151	35.4	112	50.9	257	64.9	

**No. Sex Partners (past 2 yrs)**									< 0.01
**2+**	87	35.1	137	31.0	58	25.3	29	6.8	
**1**	161	64.9	305	69.0	171	74.7	397	93.2	

**Current Birth Control Use**									< 0.01
**Yes**	34	12.7	86	19.9	48	21.2	17	4.2	
**No**	233	87.3	346	80.1	178	78.8	390	95.8	

**In Household with Co-wives**									< 0.01
**Yes**	46	16.4	220	50.1	104	45.0	183	42.8	
**No**	234	83.6	219	49.9	127	55.0	245	57.2	

**Number of Malaria Diagnoses** **(past 2 yrs)**									< 0.01
**3+**	67	26.3	126	30.5	79	35.8	181	45.6	
**2**	57	22.3	117	28.3	74	33.5	123	31.0	
**1**	102	40.0	129	31.2	46	20.8	78	19.6	
**0**	29	11.4	41	9.9	22	9.9	15	3.8	

^a^Fisher's Exact test p-value.

Table 3 shows the risk of HR-HPV infection for table 2 variables within age strata. We had hypothesized that the risk factors might help explain the high HR-HPV prevalence at older ages. However, age stratification did not reveal strong and consistent explanatory differences. The slight differences are as follows: An age of 10-14 years at sexual debut was associated with a five-fold risk for HR-HPV (10-14 years: OR 5.0; 15-24 years: OR 1.2; *p-trend = 0*.02) in women aged 30-45 years. Early age at first pregnancy was associated with a greater than two-fold risk for HR-HPV in women aged 56+ years (12-19 years: OR 2.4; 20-24 years: OR 2.2; *p-trend *= 0.04). Early age at sexual debut was also a risk factor among women in this age group, although the association was not quite as strong (15-24 years: OR 1.6; *p-value *= 0.15). Multiple sex partners and birth control use were risk factors for women aged 30-45 (OR 1.7; *p-value *= 0.08 and OR 1.8; *p-value = 0*.8, respectively). Living in a household with co-wives was not associated with HR-HPV in this age stratified analysis. We did observe somewhat of a dose response relationship with the number of recent self-reported malaria diagnoses and HR-HPV prevalence in women aged 15-29 years, although this relationship was not significant (*p-trend *= 0.31).

**Table 3:** Risk factors for high risk human papillomavirus positivity stratified by age

		15-29 Years			30-45 Years			46-55 Years			56+ Years		
		**n (%HR-HPV)**	**OR**	**95% CI**	**n (%HR-HPV)**	**OR**	**95% CI**	**n (%HR-HPV)**	**OR**	**95% CI**	**n (%HR-HPV)**	**OR**	**95% CI**

**Age of Sexual Debut (years)**	10-14	20 (25.0)	0.3	0.04 - 3.0	19 (36.8)	5.0	1.1 - 23.0	7 (0.0)	N/A	N/A	1 (0.0)	N/A	N/A
	15-24	226 (18.1)	0.2	0.03 - 1.6	365 (11.8)	1.2	0.3 - 4.0	168 (13.1)	1.1	0.4 - 3.1	241 (17.4)	1.6	0.8 - 3.1
	25+	4 (50.0)	1.0	Ref	29 (10.3)	1.0	Ref	41 (12.2)	1.0	Ref	112 (11.6)	1.0	Ref
		p-value^a ^= 0.29			p-value^a ^= 0.02			p-value^a ^= 0.90			p-value = 0.15		

**Age at First**	12-19	70 (15.7) 0.5 0.1 - 1.8			53 (13.2) 1.5 0.6 - 3.9			27 (18.5) 1.4 0.5 - 4.3			17 (23.5)	2.4	0.7 - 8.1
**Pregnancy**	20-24	99 (22.2) 0.7 0.2 - 2.5			214 (15.0) 1.7 0.9 - 3.4			77 (10.4) 0.7 0.3 - 1.8			102 (21.6)	2.2	1.2 - 4.1
**(years)**	25+	14 (28.6) 1.0 Ref			139 (9.4) 1.0 Ref			108 (13.9) 1.0 Ref			214 (11.2)	1.0	Ref
		p-value^a ^= 0.42			p-value^a ^= 0.30			p-value^a ^= 0.54			p-value = 0.04		

**No. of Sex Partners (past two years)**	2+	84 (22.6)	1.4	0.7 - 2.7	132 (17.4)	1.7	1.0 - 3.0	56 (14.3)	1.1	0.5 - 2.7	25 (16.0)	1.1	0.4 - 3.2

	1	154 (17.5)	1.0	Ref	289 (11.1)	1.0	Ref	165 (12.7)	1.0	Ref	336 (15.2)	1.0	Ref
		p-value = 0.34			p-value = 0.08			p-value = 0.77			p-value = 0.91		

**Current Birth Control Use**	Yes	33 (27.3)	1.8	0.8 - 4.1	84 (19.1)	1.8	1.0 - 3.4	48 (16.7)	1.4	0.6 - 3.4	17 (23.5)	1.8	0.6 - 5.7
	No	206 (17.5)	1.0	Ref	328 (11.6)	1.0	Ref	170 (12.4)	1.0	Ref	331 (14.8)	1.0	Ref
		p-value = 0.19			p-value = 0.08			p-value = 0.45			p-value = 0.36		

**In Household with Co-wives**	Yes	43 (18.6) 0.9 0.4 - 2.2			211 (13.7) 1.1 0.6 - 2.0			100 (13.0) 1.0 0.4 - 2.2			163 (12.3)	0.7	0.4 - 1.2

	No	200 (19.5) 1.0 Ref			208 (12.5) 1.0 Ref			121 (13.2) 1.0 Ref			200 (17.5)	1.0	Ref
		p-value = 0.89			p-value = 0.71			p-value = 0.96			p-value = 0.16		

**Number of Malaria Diagnoses (past two years)**	3+	62 (21.0)	3.2	0.7 - 15.3	121 (15.7)	1.2	0.4 - 3.6	77 (11.7)	0.4	0.1 - 1.4	153 (15.0)	1.1	0.2 - 5.1
	2	48 (22.9)	3.6	0.7 - 17.5	111 (8.1)	0.6	0.2 - 1.9	72 (11.1)	0.4	0.1 - 1.3	104 (16.4)	1.2	0.2 - 5.7
	1	86 (16.3)	2.3	0.5 - 11.0	123 (12.2)	0.9	0.3 - 2.7	43 (11.6)	0.4	0.1 - 1.6	63 (14.3)	1.0	0.2 - 5.2
	0	26 (7.7)	1.0	Ref	38 (13.2)	1.0	Ref	20 (25.0)	1.0	Ref	14 (14.3)	1.0	Ref
		p-value^a ^= 0.31			p-value^a ^= 0.35			p-value^a ^= 0.47			p-value = 0.98		

a - p-trend.

## Discussion

The main goal of this study was to identify risk factors for HR-HPV; particularly those associated with age that would help to explain the increased prevalence of HR-HPV infection among older women in this cohort. Instead, among all women, we found expected behavioral determinants of viral positivity, including early age at sexual debut and multiple sex partners. Although we did find that early age at sexual debut and first pregnancy were somewhat stronger risk factors among women aged 30-45 and 56+ years, respectively, the effects were not sufficiently different between the age groups to explain the high HR-HPV prevalence older women.

We hypothesized that being a co-wife might correlate with HR-HPV appearance at older ages since a woman in a polygamous marriage is theoretically engaged in sexual relations with more than one partner; however we did not observe this finding. We also hypothesized that women with HR-HPV at older ages might be at increased risk due to partial immunosuppression from to chronics parasitosis, however there was no association with recent self-reported malaria. Although self-reported malaria exposure was not associated with risk for HR-HPV infection, we did find an increase in recent reported malaria cases among women aged 56+ years compared to women in younger age groups. Even though it was not related to HR-HPV, this is an interesting finding given that prevailing research in endemic areas indicates the highest malaria burden to be among younger age groups [18,19]. It is possible that self-reported malaria exposure is inaccurate, attenuating our results. A recent study has shown that malaria self-report data is highly sensitive and specific when prevalence is measured over a 6-month time frame, but that specificity dramatically decreased when recalling after 18 and 30 months [20].

One important limitation of this study was the potential for selective response bias on our clinic questionnaire, particularly regarding questions related to sexual and reproductive behavior as discussions of sexuality are considered taboo among some Yoruba women [21,22]. The present study did not include HIV seroprevalence data. However, we believe that HIV is unlikely to explain the elevated HR-HPV prevalence in older women. If anything, we would have expected HIV immunosuppression to elevate HR-HPV prevalence at earlier ages. We also did not have information on the husband or partner's sexual behavior and therefore additional confounding effects cannot be ruled out. The cross-sectional nature of this study only permitted us to look at HR-HPV prevalence and therefore we were not able to evaluate risk factors for HR-HPV acquisition and persistence.

## Conclusions

The explanation for high HR-HPV prevalence at older ages in rural Nigeria and other regions of Sub-Saharan Africa remain elusive. Whether increased prevalence at older ages is due to acquisition of new infections or to attenuation in the immune response leading to a reemergence of a latent infection remains unclear [7]. Data from ongoing cohort studies have shown that the same HR-HPV type can occasionally re-appear after apparent clearance [23]. Our findings underscore the need for enhanced knowledge about HR-HPV risk factors in older women, as screening programs are not generally targeted for this age group [12]. Future studies should include more precise characterization of important health determinants such as immune markers of malaria exposure as well as more detailed information on male sexual behavior [24]. Moving beyond questionnaire data, we are now pursuing serum-based studies of general immunity and age-specific HR-HPV prevalence in the Irun population.

## Competing interests

The authors declare that they have no competing interests.

## Authors' contributions

MC: Analyzed data, drafted manuscript. JG: Involved with the design of the study, acquisition and interpretation of data, and provided critical revisions of manuscript. KA: Local investigator, contributed substantially to the acquisition of data and design of the project. NW: Involved with the conception and design of the study and provided critical revisions of the manuscript. AA: Local investigator, contributed substantially to the acquisition of data and design of the project. SW: Biostatistician, involved in the design of the study and provided critical revisions of manuscript. RB: Laboratory collaborator, performed HPV typing, and provided critical revisions of manuscript. MS: Principal Investigator, involved in the conception and design of the study, and provided critical revisions of manuscript.

All authors read and approved the final manuscript.
